# Socio-economic determinants of anemia in pregnancy in North Shoa Zone, Ethiopia

**DOI:** 10.1371/journal.pone.0202734

**Published:** 2018-08-22

**Authors:** Fantahun Ayenew Mekonnen, Yohannes Abere Ambaw, Genanew Timerga Neri

**Affiliations:** 1 Department of Epidemiology and Biostatistics, Institute of Public Health, College of Medicine and Health Sciences, University of Gondar, Gondar, Amhara Regional State, Ethiopia; 2 Institute of Medicine and Health Sciences, Bahir Dar University, Bahir Dar, Amhara Regional State, Ethiopia; 3 Department of Statistics, College of Computational and Natural Sciences, Debre Berhan University, Debre Berhan, Amhara Regional State, Ethiopia; University of Ottawa, BANGLADESH

## Abstract

**Background:**

Globally, anemia in pregnancy increases maternal, fetal and neonatal mortality and morbidity. According to 2011 Ethiopian Demographic and Health Survey, 22% of pregnant women in Ethiopia were reported to be anemic. However, since the Ethiopian population is diverse with regard to culture, religion and other characteristics, this evidence may not represent the condition in our study area. So, we aimed to determine the prevalence of anemia and its associated factors among women receiving Antenatal Care (ANC) in Debre Berhan Town Hospitals and Clinics.

**Methods:**

We conducted an institution based cross sectional study among women receiving ANC at hospitals and clinics in Debre Berhan Town, Ethiopia from September to November, 2013. Antenatal care providers in the respective health facilities collected the data by interview and observation using closed and open-ended questions. We computed frequencies and percentages to describe the data. We performed bivariate and multivariable binary logistic regression analyses to identify factors associated with anemia in pregnancy. STATA version 12 was used to carry out the analyses.

**Results:**

A total of 295 participants completed the study, with a response rate of 89%. This study demonstrated a 10% prevalence of anemia out of which 64.3%, 32% and 4% of the respondents were with mild, moderate and severe anemia respectively. Anemia was statistically significantly associated with education and occupation.

**Conclusion:**

The prevalence of anemia in our study area is lower than previous studies’ findings. Literacy and job status of the women were predictors of anemia in pregnancy. Since this study was conducted on women who had an opportunity to visit health facilities, it is more valuable to conduct community based research to better understand the problem in the study area and thus propose future deliverable.

## Introduction

Anemia occurs mainly due to iron deficiency though folic acid, vitamins A, C and B12 and other vitamins of the B-complex group i.e., niacin and pantothenic acid, proteins, and amino acids are essential in maintaining the hemoglobin level [1].

It occurs at all stages of the life cycle, but is more prevalent in pregnant women [2, 3]. A category specific prevalence study shows 41% of pregnant women worldwide were affected compared to the prevalence among non-pregnant women (30.2%), school age children (25.4%), elderly (23.9%) and men (12.7%) [3]. Anemia in pregnancy occurs when the hemoglobin level in the blood is below 11gm percent [3].

Anemia increases the maternal morbidity, fetal and neonatal mortality and morbidity [2, 4]; About 4–16% of maternal deaths are due to anemia [5]. Anemia in late pregnancy results in poor fetal iron stores; latent iron deficiency is known to irreversibly alter brain iron content and neurotransmitters in fetal life and postnatal babies [5–7].

The predisposing factors of anemia in pregnancy are diverse ranging from the individuals’ behaviors to community characteristics. Socioeconomic and cultural, nutrition, parasitic diseases like malaria and hookworm, and human immune deficiency virus infection are the most understood factors responsible for its occurrence [8].

According to 2011 Ethiopian Demographic and Health Survey report, 22% of Ethiopian pregnant women were anemic [9]. However, since Ethiopia is a country with diverse population with regard to culture, religion and other important characteristics, this evidence may not reflect the conditions in specific areas of the country. For instance, a study conducted in Jimma, western Ethiopia revealed a prevalence of 38% [10] which is significantly different from the nationwide prevalence. To our knowledge, however, no research has been conducted in North Shoa Zone, Central Ethiopia to determine the extent of the problem and hence propose recommendations of policy or further research implications. So, we aimed at finding out the level of anemia in pregnancy and its associated factors in North Shoa, Ethiopia.

## Methods and materials

### Study design and setting

We conducted a cross sectional study among mothers receiving ANC at health institutions in Debre Berhan Town, North Shoa, Ethiopia from September to November, 2013. All health institutions in the town were included in the study. The institutions were two hospitals (one government and another private), and four clinics out of which one was private clinic and the other one was a clinic run by a charity organization while the rest two were government clinics.

### Sample size and procedure

The sample size was calculated by single population proportion formula using a prevalence of anemia (38.2%) from a previous study conducted at a referral hospital in Jimma, Ethiopia in 2005 [10], a confidence level of 95% and a margin error of 5%. The sample size was 333 including a 10% non-response rate. Pregnant women receiving ANC during the study period and who met the inclusion criteria were recruited. A sample proportional to each institution’s total number of women of ANC attendant in the previous one month was allocated to each health facility.

### Data collection

Questionnaire with closed and open-ended questions was used to collect data on: socio-economic and socio-demographic characteristics; maternity characteristics; eating and nutrition related characteristics; infections; and knowledge and attitudes about anemia. Data related to blood examination results of infections status and hemoglobin (Hgb) level, and height and weight were, collected from the respondent’s current charts. The nutrition related characteristics of respondents specified by frequency and amount, included food items like meat and green vegetables or fruits consumption history. On the other hand, maternity variables were previous history of pregnancy and birth, number of pregnancies, birth spacing, and age at first pregnancy. Disease and infection status included the commonly reported infections in various literatures such as HIV and hock worm infection, and malaria diseases history. We also included variables describing previous visit of health facilities or use of maternal services like delivery, ANC or family planning. Nurses who were able to speak the local language and who provided ANC services interviewed the women waiting for their turn for the ANC service, and then they reviewed the charts immediately after the women received the services. They collected the data after they obtained training on data collection procedures to maximize inter-interviewer reliability. The data collectors were regularly supervised by the principal investigators to ensure reliable data collection.

### Data processing and analysis

The data completeness and consistency were checked, and then the variables were coded by the principal investigators, and entered in to epi-info version 2002 software, and exported to STATA version 12 for further analyses. We described the data by computing frequencies, summary measures and graphs to understand the nature of the data. Later, we employed a binary logistic regression model to perform both the bivariate and multivariable analyses. Each predictor variable was examined for the presence of statistical association with the dependent variable using the bivariate regression model. Predictor variables with a p-value of 0.2 or below in the bivariate analyses were taken to the multivariable logistic regression model to assess its independent effect on anemia in pregnancy. Independent variables having a p-value of less than 0.05 in the multivariable analysis were considered as statistically significantly associated with the dependent variable. We assessed the final model for its fitness to the data by Hosmer and Lemeshow’s goodness of fit test. The model was also assessed for the presence of multi-collinearity, interaction and confounding. Both crude and adjusted odds ratios with their 95% confidence intervals were extracted from the final models.

### Ethical consideration

Ethical approval was obtained from Debre Berhan University Institute of Medicine and Health Sciences Research Ethics Review Committee. A letter of cooperation was written to Debre Berhan town health administration office by the university. Permission was obtained from the respective hospitals and clinics managers to approach the study participants. Finally, informed oral consent was obtained from each participant for interview and accessing laboratory examination results. During the actual data collection, we closely supervised the data collectors to ensure respect of the fundamental research ethics principles and rules, which included informed decision for participation, privacy and confidentiality.

## Results

### Descriptive results

A total of 295 study participants completed the study with a response rate of 89%. The median age of the participants was 25 with an inter quartile range of 7 years. Over two-thirds, 228 (78%), were between 15 and 29 years of age. Most, 266 (92%), of the women were married. Of the educational status reported, elementary schooling was the modal, 102 (35%), education level attended, while 23 (8%) were women who were able to read and write only. Concerning occupation, over half, 71 (58%), of the women were house wives, while 50 (17%) were employed in either government or non-governmental organizations. With regard to residence and monthly family income, 193 (68%) were urban dwellers, and 120 (41%) were less than $24 monthly income earners.

Approximately two-thirds, 185 (63%) of women had a previous history of using family planning. Just over half, 158 (54%), of the respondents reported one or more previous pregnancies. The first pregnancy occurred at < 30 years of age for the majority, 240 (94%), of the respondents. Forty-four percent of the respondents were at their third trimester at the time of data collection. Nearly fifty two percent of women reported nausea and vomiting during their current pregnancy.

Regarding eating habits, over half, 166 (57.24%), ate green vegetables once a week, and most, 229 (78.42%), reported eating meat occasionally. Below half, 126 (44%), drank tea regularly. The body mass index of 206 (71%) women were between 20–24.99kg/m^2^, while 4(1%) were between 30-40kg/m^2^.

Of the 295 participants whose blood was examined, 6 (2%) were HIV positive. History of malaria infection was reported by 28 (10%), but no current infection was identified.

We identified anemia from 28 (9.7%) of the women visiting ANC centers; 18 (64.29%) were classified as mild, 9 (32.14%) were classified as moderate, and 1 (3.57%) was severe anemia. (Table 1)

**Table 1:** Characteristics of women receiving ANC at health institutions in Debre Berhan Town, Ethiopia from Sept to Nov, 2013.

Characteristics	Frequency	percent
***Socio-demographic characteristics***		
Age (years)		
15–29	228	77.55
30–49	66	22.45
Marital status		
Married	266	91.72
Single	24	8.28
Educational status		
Unable to read & write	57	19.39
Read & write only	23	7.82
Elementary school	102	34.69
Nine–Twelve grade	53	18.03
Certificate and above	59	20.07
Occupational status		
Employed	50	16.95
Private owner	51	17.29
House wife	171	57.97
Others	23	7.80
Residence		
Urban	193	67.72
Rural	92	32.28
Monthly Income(USD)		
< 24	120	41.38
24–39.9	59	20.34
≥40	111	38.28
***Maternal characteristics***		
Use family planning methods		
Yes	185	62.93
No	109	37.07
Previous Pregnancy		
Yes	158	54.48
No	132	45.52
Age of current pregnancy		
< 3 months	78	26.71
3–6 months	87	29.79
>6 months	127	43.49
***Eating/ nutritional status***		
Eat green vegetables		
Daily	26	8.97
Two-Five times a week	38	13.10
Once a week	166	57.24
Occasionally	60	20.69
Eat meat		
Daily	10	3.42
Two-Five times a week	18	6.16
Once a week	35	11.99
Occasionally	229	78.42
Drink tea		
Yes	162	56.25
No	126	43.75
Body Mass Index(kg/m^2^)		
≤ 19	50	17.24
20–24.99	206	71.03
25–29.99	30	10.35
30–40	4	1.38
***Infections/diseases/disorders***		
Nausea & vomiting		
Yes	149	52.10
No	137	47.90
Malaria infection		
Yes	28	9.69
No	261	90.31
HIV infection		
Yes	6	2.17
No	271	97.83
Anemia status		
Hgb> = 11g/dl	262	90.34
Hgb = 9–10.9g/dl	18	6.21
Hgb = 7–8.9 g/dl	9	3.10
Hgb< 7g/dl	1	0.35

### Factors associated with anemia in pregnancy

We examined variables believed to cause differences in developing anemia in pregnancy according to previous evidences and experiences, for possible associations with anemia status using binary logistic regression model. Firstly, we performed a bivariate logistic regression analysis to identify predictor variables associated with anemia in pregnancy. Independent variables, which included nausea and vomiting in the current pregnancy, average monthly income, residence, education and occupation of the women were identified to have a p-value less than 0.2 in the bivariate analysis. These variables were then taken to the multivariable binary logistic regression model to see if there were yet variables associated with anemia in pregnancy. In the multi variable analysis, we identified occupation and education characteristics of the women to be statistically significantly associated with anemia at 5% level of significance yielding a Hosmer and Lemeshow’s goodness of fit test p-value of 0.79. The direction of the association between educational status and anemia in pregnancy, however, seemed to be the reverse of what should have been according to the knowledge and experience of the scientific community. Regarding women’s occupation, house wives and private workers were found to be more protected compared to women employees. (Table 2)

**Table 2:** Factors associated with anemia among women receiving ANC at health institutions in Debre Berhan Town, Ethiopia from Sept to Nov, 2013.

Anemia status			
Characteristics	Yes No (%)	No No (%)	AOR(95%CI), P-value
Educational status			0.02
Unable to write and read	3(10.71)	52(19.92)	1
Read and write only	2 (7.14)	21(8.50)	1.05(0.15, 7.56)
Elementary	9(32.14)	91(34.87)	1.77(0.45, 6.99)
Secondary	10(35.71)	43(16.48)	4.50(1.12, 18.13)
Certificate and above	4(14.29)	54(20.69)	0.27(0.04, 2.09)
Job status			0.018
Employed	7(25.00)	42(16.03)	1
Private owner	2(7.14)	49(18.70)	0.05(0.01, 0.36)
House wife	15(53.57)	152(58.02)	0.14(0.03, 0.66)
Other	4(14.29)	19(7.25)	0.28(0.04, 1.73)

## Discussion

The study was aimed at estimating the prevalence of anemia in pregnancy and identifying its associated factors among women receiving ANC at health institutions in Debre Berhan Town, Ethiopia. This study, revealed a 9.7% (95% CI; 6%-13%) overall prevalence of anemia in pregnancy. Women’s education and job status were identified statistically significantly associated with anemia in pregnancy. Of the 9.7% of women with anemia, 64% of our respondents had mild anemia, 32% were moderately anemic and 4% had severe anemia. The overall prevalence of anemia is our study is lower than the finding of a study conducted in Jimma, Ethiopia (38%) and Ethiopian national survey report (22%) [9, 10]. It is also lower than the prevalence in Tanzania (47%), Mali (47%) and Nigeria (40%) [11–13] along with other countries including Pakistan (91%), India (75%), Turkey (43%) and China (26%) [14–17]. The inconsistency between this study finding and the finding in Jimma might be because that study included only first visitors of ANC for the current pregnancy in which it is normal to see an excess number of pregnant women with anemia in such populations. This is due to the fact that women who repeatedly visit health facility are those who are with better awareness on health and thus with lesser chance of developing health risks [18]. On the other hand, the low prevalence of anemia observed in our study compared to the Ethiopian national survey report could be attributed in part to the study population and/or the study setting difference. Meaning, we studied health facility visitors; while the national survey was community based research which can reach the most deprived members of the community in their natural setting resulting in an elevated overall anemia prevalence in pregnancy. A given population’s culture, norms and religion are important aspects in causing differences in eating and nutrition, making that group of population as a whole to be more at risk compared to other population groups with opposing characteristics [19]. Ethiopian population is diverse in many aspects which can affect eating, health care utilization that in turn call varied risks of anemia among the different population groups. Thus, though the national survey was carried out in such a way that the finding can reflect the situation of the problem in the Ethiopia population in general, it is unfair to apply a prevalence finding to a specific homogenous group of population. Similar reasons might have played a role in creating a big discrepancy in anemia prevalence between our finding and the findings reported from different countries mentioned above apart from effects coming from variation in race and socio economic status. For instance, the high prevalence of anemia in Is Parta, Turkey might be due to the fact that thalassemia is highly prevalent in Turkey [19], while in India it might be in part because that study population was purely rural population [15].

With regard to the factors responsible for developing anemia in pregnancy, a couple of variables which were job and literacy of the pregnant women were identified associated with anemia in pregnancy in the multivariable analysis. Previous studies revealed that education of women was statistically significantly associated with anemia occurrence among pregnant women [10, 20].

Over all, the prevalence of anemia among pregnant women in the current study was found to be 9.7% which is lower than previous studies. We identified Literacy and job status of the women significantly associated with anemia status. But, the direction of the association between the education status of women and anemia in pregnancy was against what should have been according the available knowledge. Since this study was conducted on women who had the opportunity to visit health facilities, it is more valuable to conduct community based research to better understand the problem in North Shoa Zone and thus propose future deliverables. We also suggest analytical study to investigate the determinants of anemia in pregnancy. We further recommend verification of the direction of association demonstrated between educational status of women and anemia in pregnancy specifically.

## Supporting information

S1 Quest This is the English version of the questionnaire. (DOCX)

S2 Quest This is the Amharic version of the questionnaire. (DOCX)

S1 Data This is the data set of the study. (DTA)
