# Supplementary material for: Socio-economic determinants of anemia in pregnancy in North Shoa Zone, Ethiopia
Source: PLoS One. 2018 Aug 22;13(8):e0202734. doi: 10.1371/journal.pone.0202734 (PMC6105028; doi:10.1371/journal.pone.0202734)
Supplement: S1 Quest — (DOCX) [file pone.0202734.s001.docx]

English Version Questionnaire Prepared to gather anemia information on women attending ANC at Health institutions in Debre Berhan Town on their anemia status and other characteristics.

Introduction:

Hello! My name is ______________ .I am collecting data for a study conducted on pregnant women attending ANC at health institutions in Debre Berhan town to identify the number of women with anemia and contributing factors by Debre Berhan University researchers. So, you are one of the study participants from whom I will be collecting information. The information comprises your socio demographic, previous family planning methods utilization, pregnancy, delivery, eating behavior, infections and anemia status. The information that you will give us will be kept confidential and will not be transferred to the third body. Your name will not appear on the questionnaire. Rather codes will be used to represent the identifiers. You will not be benefited because you are willing nor will be harmed because are not willing to participate. But, the result of this study will be helpful in improving women’s and children’s health. No one will deprive you from receiving the ANC services available whether you are willing or not. The interview will take 10-15minuts of your time. If you feel discomfort at any time you can discontinue or interrupt the interview.

Are you willing?

If yes, continue asking the questions below.

If no, skip this participant and proceed to other participant.

**Part one: Socio-demographic characteristics**

| S.no | Questions | Response options |
| --- | --- | --- |
| 101 | Age | ____years |
| 102 | What is your ethnicity? | 1. Amhara 2. Oromo 3. Tigrie 4. Other (specify)_____ |
| 103 | What is your religion | 1. Orthodox 2. Muslim 3. Protestant 4. Others (specify)____­ |
| 104 | What is your education level? | 1. Unable to read and write 2. Read and write only 3. 1-4^th^ grade 4. 5-8^th^ grade 5. 9-12^th^ grade 6. Certificate 7. Diploma and above |
| 105 | What is your job status? | 1. Governmental organization employee 2. Non-governmental organization employee 3. Farmer 4. House wife 5. Jobless 6. Other (specify)_____ |
| 106 | What is your marital status? | 1. Married 2. Single 3. Divorced 4. Widowed 5. Separated |
| 107 | Where is your residence? | 1. Urban 2. Rural |
| 108 | What is the household average monthly income? | _________Ethiopian Birr |
| 109 | Relationship to the house leader? | 1. Wife 2. Daughter 3. Servant 4. Other (specify)______ |
| 110 | What is the household family size? | ____________ |

**Part two: Maternity characteristics**

| S.no | Questions | response |
| --- | --- | --- |
| 201 | Have been pregnant in the past | 1. Yes 2. No |
|  | **If the answer for question no 201 is yes, go to question no 211-217** |  |
| 202 | How many pregnancies have you had excluding the current pregnancy? | _________ |
| 203 | Have you had ANC follow up for at least one of the past pregnancies? | 1. Yes 2. No |
| 204 | What was the outcome of the nearest pregnancy? | 1. Normal full term birth 2. Preterm 3. Still birth 4. Aborted |
| 205 | Do you have a child currently? | 1. Yes 2. No |
| 206 | If the answer is yes, how many children do you have? | ___________ |
| 207 | What was the space between the current pregnancy and the previous most recent birth? | 1. Less than one year 2. One-two years 3. Three and above years |
| 208 | Have ever delivered at health facility | 1. Yes 2. No |
| 209 | Where did you deliver your most recent birth? | 1. Health facility 2. Home |
| 210 | If at home, who assisted the delivery? | 1. Trained health professionals 2. Trained community birth attendants 3. Traditional birth attendant 4. Family |
| 211 | Have you experienced bleeding in the past 2weaks? | 1. Yes 2. No |
| 212 | At what gestational age is the current pregnancy? | 1. Below three months 2. Three-six months 3. Over six months |
| 213 | What is the total number of ANC visit you have for the current pregnancy? | 1. This is the first 2. Two times 3. Three times 4. Four times and above |
| 214 | Have you ever used family planning methods? | 1. Yes 2. No |
| 215 | If your answer is yes, which family planning method you have used? | 1. Traditional family planning 2. Modern family planning |
| 216 | If it is modern family planning, which specific method you have used in your recent practice? | 1. Condom 2. Pills 3. Injectible 4. Implants 5. Intra uterine device 6. Other (specify)_______ |
| 217 | What was your age at your first pregnancy? | __________________ |

**Part three: Knowledge and attitude about anemia**

| s.no | Questions | Response options |
| --- | --- | --- |
| 301 | Have you ever heard of what anemia means? | 1. Yes 2. No |
| 302 | If yes, where did you get it? | 1. Health professionals 2. Mass media 3. Family 4. Neighbors 5. Other (specify)________ |
| 303 | What do think are the causes of anemia? | ___________________________________ |
| 304 | Do think that anemia in pregnancy is preventable? | 1. Yes 2. No |
| 305 | If yes, how it can be prevented? | ____________________________________ |
| 306 | Do you think that pregnancy can result in anemia? | 1. Yes 2. N0 |
| 307 | Do you think that anemia can be treated? | 1. Yes 2. N0 |
| 308 | Will you take medicine if you have anemia | 1. Yes 2. No |
| 309 | Have you ever thought that you may develop anemia? | 1. Yes 2. No |
| 310 | *If your answer is no, please mention the reasons* | ______________________________  _________________ |
| 311 | Do you accept health professional’s nutritional advice? | 1. Yes 2. No |
| 312 | Do think that birth spacing can minimize the chance developing anemia? | 1. Yes 2. No |
| 313 | Do you change your eating habit when you become pregnant? | 1. Yes 2. No |
| 314 | If yes, what type of eating habit you adapt? | 1. Increasing the amount of food 2. Cut the amount of food 3. Changing the type of food 4. Other (specify)_______ |

**Part four: Eating and nutrition**

| S.no | Questions | Response option |
| --- | --- | --- |
| 401 | What is the frequency of eating at least one of these foods items? (Meat, milk or egg) | 1. Daily 2. Two–Five times a week 3. Once per week 4. occasionally |
| 402 | What is the frequency of eating green vegetables? | 1. Daily 2. Two–Five times a week 3. Once per week 4. occasionally |
| 403 | Do you drink tea? | 1. Yes 2. No |
| 404 | If yes, how many times per day you drink tea? | ______________ |
| 405 | Do you drink coffee? | 1. Yes 2. No |
| 406 | If yes, how many times per day you drink coffee? | _________ |
| 407 | How much is the respondent’s weight? | _________kg |
| 408 | How much is the respondent’s height? | _________Meter |

**Part five: Infection/disease/disorder related information**

| **s.no** | **Questions** | **Response option** |
| --- | --- | --- |
| **501** | Are you experiencing nausea and vomiting in the current pregnancy? | 1. Yes 2. No |
| **502** | Have you had malaria? | 1. Yes 2. No |
| **503** | What is the hook worm infection test result? | 1. Positive 2. Negative |
| **504** | What is HIV/AIDs infection test result? | 1. Positive 2. Negative |
| **505** | What is the hemoglobin level? | 1. Hgb > 11g/dl  3. Hgb = 7-8.9g/dl  2. Hgb = 9-10.9g/dl  4. Hgb < 7g/dl |

I would like to thank you for your time!
