# Supplementary material for: Socio-economic determinants of anemia in pregnancy in North Shoa Zone, Ethiopia
Source: PLoS One. 2018 Aug 22;13(8):e0202734. doi: 10.1371/journal.pone.0202734 (PMC6105028; doi:10.1371/journal.pone.0202734)
Supplement: S2 Quest — (DOCX) [file pone.0202734.s002.docx]

**መግቢያ**

እኔ­­­­­­­­­---------------እባላለሁ፡፡ ይህ በደብረ ብርሃን ዩኒቨርሲቲ የእናቶችን የደምማነስ (anemia) ሁኔታን ለማጥናት የተዘጋጀ መጠይቅ ሲሆን እርስዎም በዚህ መጠይቅ ላይ ይሳተፉ ዘንድ በአክብሮት ጋብዘንዎታል፡፡ የጥናቱ አላማም በደብረ ብርሃን ከተማ ውስጥ በሚገኙ የጤና ተቆማት ለእርግዝና ክትትል ከሚመጡት እናቶች ውስጥ የደም ማነስ (anemia) ችግር ያለባቸዉን እናቶች እና መንስኤዎቻቸውን በመለየት የመፍትሄ አቅጣጫዎችን ለሚመለከታቸዉ አካላት መጠቆም ነዉ፡፡ሥለዚህ የዚህ ጥናት አላማ ይሳካ ዘንድ እርስዎ በዚህ ጥናት እንዲሳተፉ በአክብሮት እንጠይቅዎታለን፡፡ በጥናቱ እንዲሳተፉ አይገደዱም፡፡ መጠይቁ 10-15 ደቂቃ የሚሆንውን ጊዜዎትን ሊወስድብዎት ይችላል፡፡በመጠይቁ ውስጥ የእርስዎን ማንነት የሚገል ስምም ሆነ ሌላ ገላጭ ነገር የለም፡፡እርስዎ የሚሰጡን መረጃ ሚስጥራዌነቱ የተጠበቀ ይሆናል፡፡ መረጃው ከዚህ ጥናት ውጭ ለሌላ አላማ አይውልም ወይም ለሌ ጥቅም አይዉልም፡፡

ፈቃደኛ ነዎት? አዎን ከሆነ የሚከተሉትን ጥያቄወች ይጠይቁ፡፡ አይደለሁም ከሆነ ወደሚቀጥለው ተሳታፊ ይቀጥሉ፡፡

**ክፍል አንድ፡ የማህበራዊ እና የኢኮኖሚያዊ ሁኔታን የሚመለከት ጥያቄ**

| **ተ.ቁ** | **ጥያቄ** | **አማራጭ መልስ** |
| --- | --- | --- |
| **101** | ዕድሜ | --------------------- |
| **102** | ብሄረሰብ | 1. አማራ 2. ኦሮሞ 3. ትግሬ 4. 4. ሌላ (ይጥቀሱ)-------------- |
| **103** | ሃይማኖት | 1. ኦርቶዶክስ  2. ሙስሊም  3. ፕሮቴስታንት  4. ሌላ (ይጥቀሱ)-------- |
| **104** | የት/ት ደረጃ | 1. ማንበብና መጻፍ የማትችል 2. ማንበብና መጻፍ ብቻ የምትችል 3. 1—4 ክፍል  4. 5-8 ክፍል 5. 9—12 ክፍል  6. ሰርተፍኬት  7. ዲፕሎማ እና ከዚያ በላይ |
| **105** | የስራ ሁኔታ | 1. የመንግስት ሰራተኛ 4. የቤት እመቤት  2. መንግስታዊ ያልሆነ ድርጀት ሰራተኛ 5. ስራ የሌለው (ስራ አጥ)  3. የግል ስራ ሰራተኛ 6. ልላ(ይጠቀስ)------------  6. ሌላ (ይጥቀሱ)------------ |
| **106** | የጋብቻ ሁኔታ | 1. ያገባች 2. ያላገባች   3. የፈታች  4.ባልዋ የሞተባት  5. ተለያይተው የሚኖሩ |
| **107** | የመኖሪያ አድራሻ | 1. ከተማ  2. ገጠር |
| **108** | አማካይ የወር ገቢ | -----------------------------------ብር |
| **109** | በቤተሰብ ውስጥ የዝምድና ሁኔታ | 1. እናት   2. ልጅ  3. ተቀጣሪ ሰራተኛ  4. ሌላ (ይጥቀሱ)-------- |
| **110** | በቤት ውስጥ የሚኖሩ የቤተሰብ ብዛት ምን ያክል ነው? | ­----------------- |

**ክፍል ሁለት፡ የስነ-ተዋልዶ ሁኔታን የተመለከተ ጥያቄ**

| **ተ.ቁ** | **ጥያቄ** | | **አማራጭ መልስ** |
| --- | --- | --- | --- |
| **201** | ከአሁኑ እርግዝና በፊት አርግዘው ያውቃሉ? | | 1. አዎን  2. አርግዜ አላውቅም |
| **ለጥያቄ ቁጥር 201 መልሱ የለም ከሆነ ከጥያቄ 211 እስከ 217 ያሉትን ጥያቄዎች ይጠይቁ፡፡** | | | |
| **202** | የአሁኑን እርግዝና ሳይጨምር ስንት ጊዜ አርግዘው ያውቃሉ? | ---------------------------- | |
| **203** | ከዚህ እርግዝና በፊት ለነበሩት እርግዝናዎች ለአንድ ጊዜም ቢሆን የእርግዝና ክትትል አድርገው ያውቃሉ? | 1. አዎን  2. አድርጌ አላውቅም | |
| **204** | ከአሁን እርግዝና ቀድሞ የነበረው ጽንስ ወሊድ ምን  አይነት ነበር? | 1. ጤነኛ በጊዜዉ የተወለደ  2. ጊዜው ሳይደርስ በህወት የተወለደ  3. ሞቶ የተወለደ  4. ዉርጃ | |
| **205** | ለጅ አልዎት? | 1. አዎን   2. የለኝም | |
| **206** | መልስዎ አዎን ከሆነ ስንት ልጆች አልዎት? | ---------------- | |
| 207 | ከዚህ በፊት ልጅ ከወለዱ በአሁኑ እና  በቀደመው ልጅ መካከል ያለው የጊዜ  ልዩነት ምን ያክል ነው? | 1. ከአንድ አመት በታች  2. ከአንድ እስከ ሁለት ዓመት  3. ከሶስት አመት በላይ | |
| 208 | ከጤና ተቋም ወልደው ያውቃሉ? | 1. አዎን  2. አላውቅም | |
| 209 | ከአሁኑ እርግዝና ቀደሞ የተወለደውን ልጅ የት  ወለዱት? | 1. ከቤት  2. ከጤና ተቋማት | |
| 210 | መልስዎ ከቤት ከሆነ ማን አዋለድዎት? | 1. የሰለጠኑ የጤና ባለሙያዎች  2. የሰለጠኑ የባህል አዋላጆች  3. በቤተሰብ  4. ያልሰለጠኑ የመንደር አዋላጆች  (ልምድ አዋላጆች) | |
| 211 | ከዚህ እርግዝና በፊት ከፍተኛ የወር አበባ መፍሰስ  አጋጥምዎት ያዉቃል? | 1. አዎን   2. አላጋጠመኝም | |
| 212 | የአሁኑ እርግዝና (ጽንስ) እድሜው ስንት ነው? | 1. ከሶስት ወር ያነሰ  2. 3—6 ወር  3. ከስድስት ወር በላይ | |
| 213 | የአሁን እርግዝና ክትትል ስንተኛዎት ነው? | 1. የመጀመሪያየ   1. ሁለተኛዬ 2. ሶስተኛዬ 3. ከሶስት ጊዜ በላይ | |
| 214 | የወሊድ መቆጣጠሪያ አገልግሎት ተጠቅመው ያውቃሉ? | 1. አዎን  2. ተጠቅሜ አላውቅም | |
| 215 | 2 መልሰዎ አዎን ከሆነ ምን አይነት የወሊድ  መቆጣጠሪያ ዘዴ ተጠቅመዋል? | 1. ባህላዊ እና ተፍጥሮዌ ዘዴ   2. ዘመናዊ ዘዴ | |
| 216 | መልሱ ዘመናዊ ዘዴ ከሆነ የትኛው ዘዴ ነው? | 1. ኮንዶም  2. እንክብሎች  3. በመርፌ የሚሰጥ  4. በክንደ ቆዳ ስር የሚቀበር  5. በማህፀን ስር የሚቀመጥ  6. ሌላ (ይጥቀሱ)_______ | |
| 217 | 2 የመጀመሪያ ልጅሽን ስታረግዝ እድሜሽ ስንት ነበር? | __________ | |

**ክፍል ሶስት፡ እውቀት፤ክህሎትን እና አመለካከትን የሚመለከት ጥያቄ**

| **ተ.ቁ** | **ጥያቄ** | **አማራጭ መልስ** |
| --- | --- | --- |
| **301** | የደም ማነስ ማለት ምን ማለት እነደሆነ ሰምተዉ ያዉቃሉ? | 1. አዎን  2. ሰምቸ አላዉቅም |
| **302** | አዎን ከሆነ ከየት ሰሙ? | 1. ከጤና ባለሙያዎች  2. ከመገናኛ ብዙሃን  3. ከቤተሰብ  4. ከጉረቤት  5. ሌላ(ይጠቀስ)--------- |
| **303** | የደም ማነስ እነዲከሰት መንስኤ የሚሆኑ ነገሮች ምን ይመስልዎታል? |  |
| **304** | የደም ማነስ በሽታን እንዳይከሰት መከላከል ይቻላል ብለዉ ያስባሉ? | 1. አዎን  2. አይቻልም |
| **305** | መልሱ አዎን ከሆን እንዴት መከላከል የሚቻል ይመስልዎታል? | **______________________________________________________________________** |
| **306** | ማርገዝ ለደም ማነስ የሚያጋልጥ ይመስልዎታል? | 1. አዎን 2. አይመስለኝም |
| **307** | የደም ማነስ በመታከም የሚድን ይመስልዎታል? | 1. አዎን  2. አይመስለኝም |
| **308** | የደም ማነስ ችግር ቢኖረብዎት የማከሚያ ክኒን ይወስዳሉ? | 1. 1. አዎን 2. 2. አልወስድም |

| 1. አዎን  2. አይመስለኝም | የደም ማነስ ችግር ሊኖርብኝ ይችላል ብለዎ ያስባሉ? | 309 |
| --- | --- | --- |
| __________________________________________________________________ | መልስዎ አይመስለኝም ከሆን ለምን? | 310 |
| 1. አዎ  2. አልቀበልም | ስለአመጋገብዎ የጤና ባለሙያዎን ምክር ይቀበላሉ? | 311 |
| 1. አዎን  2.አይቻልም | አራርቆ በመዉለድ የደም ማነስ ተጋላጭነትን መቀነስ የሚቻል ይመስልዎታል? | 312 |
| 1.አወን  2. አላደርግም | በሚያረግዙበት ጊዜ ከሌላወ ጊዜ በተለየ የአመጋገብ ለወጥ ያደርጋሉን? | 313 |
| 1. የምግቡን መጠኑን በመጨመር 2. የምግቡን መጠኑን በመቀነስ 3. የምግቡን አይነቱን በመቀየር 4. ሌላ(ይጠቀስ)__________________ | አዎን ከሆነ የአመጋገብ ለወጥ የሚያደርጉት በምን መልኩ ነዉ? | 314 |

**ክፍል አራት: የአመጋገብ ሁኔታን የሚመለከት ጥያቄ**

| **401** | የእንስሳት ወጤት ምግቦችን እን (ስጋ እንቁላልና የወተት ውጤቶች ቢያንስ አንዱን ስንት ጊዜ ይጠቀማሉ)? | 1. በየቀኑ  2. በሳምንት ከሁለት- አምስት ጊዜ  3. በሳምንት አንድ ጊዜ  4. እንደ አጋጠመኝ |
| --- | --- | --- |
| **402** | 3 አረንጓዴማ ቅጠል አትክልቶችን ስንት ጊዜ ይጠቀማሉ? | 1. በየቀኑ  2. በየሁለት ቀኑ  3. በሳምንት አንድ ጊዜ  4. በሁለት ሳምንት አንድ ጊዜ  5. ሌላ(ይጥቀሱ)________ |
| **403** | ቡና ይጠጣሉ? | 1. አዎን  2. አልጠጣም |
| **404** | ለጥያቄ ቁ 4.17 መልሱ አዎን ከሆነ በቀን ስንት ጊዜ ይጠጣሉ? | _____________________________ |
| **405** | ሻይ ይጠጣሉ? | 1. አዎን  2. አልጠጣም |
| **406** | ለጥያቄ ቁ 4.19 መልሱ አዎን ከሆነ በቀን ስንት ጊዜ ይጠጣሉ? | ___________________________ |
| **407** | ክብደታቸዉ ስንት ነው? | ___________ ኪሎግራም |
| **408** | ቁመታቸው ስንት ነው? | ___________ ሜትር |

**ክፍል አምስት: የህመም ሁኔታን የተመለከተ ጥያቄ**

| 501 | በመጀመሪያወቹ የአሁኑ የእርግዝና ወራት ከፍተኛ የሆነ የማቅለሽለሽ እና የማስታወክ ችግር 1ገጥሞዎት ያውቃል? | 1.አዎን  2. አላጋጠመኝም |
| --- | --- | --- |
| 502 | በወባ በሽታ ተይዘው ያውቃሉ? | 1. አዎን  2. ተይዜ አላውቅም |
| 503 | የሁክ ወረም ኢንፌክሽን የምርምራ ውጤታቸው ምን ይመስላል? | 1. ነገቲቨ  2. ፖዘቲቨ |
| 504 | የኤች አይ ቪ ኤድስ የምርመራ ሁኔታቸው ምን ይመስላል? | 1. ነገቲቭ  2. ፖዘቲቭ |
| 505 | የደም ማነስ ሂሞግሎቢን መጠናቸው ምን ያክል ነው? | 1. Hgb > 11g/dl  2. Hgb = 9-10.9g/dl  3. Hgb = 7-8.9g/dl  4. Hgb < 7g/dl |

ለጊዜዎት እጅግ በጣም አመሰግናለሁ፡፡
